# First evidence of European eels exiting the Mediterranean Sea during their spawning migration

**DOI:** 10.1038/srep21817

**Published:** 2016-02-24

**Authors:** Elsa Amilhat, Kim Aarestrup, Elisabeth Faliex, Gaël Simon, Håkan Westerberg, David Righton

**Affiliations:** 1 Univ. Perpignan Via Domitia, Centre de Formation et de Recherche sur les Environnements Méditerranéens, UMR 5110, F 66860, Perpignan, France; 2 CNRS, Centre de Formation et de Recherche sur les Environnements Méditerranéens, UMR 5110, F 66860, Perpignan, France; 3 Technical University of Denmark, National Institute of Aquatic Resources, Silkeborg, DK-8600, Denmark; 4 Swedish University of Agricultural Sciences (SLU), Institute of Freshwater Research, Drottningholm, SE-178 93, Sweden; 5 Centre for Environment, Fisheries and Aquaculture Science (CEFAS), Lowestoft, NR33 0HT, United Kingdom

## Abstract

The migration route and the spawning site of the European eel *Anguilla anguilla* are still uncertain. It has been suggested that the Mediterranean eel stock does not contribute to spawning because there is no evidence of eels leaving the Mediterranean Sea. To test this hypothesis, we equipped eight female silver eels from the south of France with pop-up satellite tags during escapement from coastal waters. Once in deeper water, the eels quickly established diel vertical migration (DVM) between the upper and lower mesopelagic zone. Five tagged eels were taken by predators within the Mediterranean, but two eels reached the Atlantic Ocean after six months and at distances greater than 2000 km from release. These eels ceased their DVM while they negotiated the Gibraltar Strait, and remained in deep water until they reached the Atlantic Ocean, when they recommenced DVM. Our results are the first to show that eels from Mediterranean can cross the Strait of Gibraltar and continue their migration into the Atlantic Ocean. This finding suggests that Mediterranean countries, as for other EU states, have an important role to play in contributing to conservation efforts for the recovery of the European eel stock.

Although European eels are found in fresh and brackish waters throughout Europe and North Africa, they escape to sea to spawn towards the end of their lives. European eels, like American eels, are presumed to spawn in the Sargasso Sea, where eel larvae of both species are found in the greatest abundance^1^,^2^,^3^. The evidence for a single spawning area is strong and modern genetic studies indicate a panmictic species^4^,^5^. European eels therefore need to undertake a spawning migration of at least 5000 km from the western margin of the continental habitat in Europe; this distance is much greater for eels escaping from more easterly locations, including those in the Baltic and Mediterranean Seas. However, despite the advent of marine remote sensing, genetic and otolith analyses, electronic tagging, and investigative computer modelling in the century since Schmidt^3^ identified the presumed spawning area, many fundamental questions about eel migrations remain unanswered.

One such question concerns the ability of eels to escape the Mediterranean Sea and contribute to the spawning stock. Ekman^6^ suggested that eels are unable to escape from the Mediterranean Sea. Their proposed halotactic and thermotactic navigational mechanisms would indeed prevent them from reaching the Atlantic Ocean^7^. Even if eels do manage to navigate westwards across the Mediterranean Sea, they must then pass through the Strait of Gibraltar to the Atlantic Ocean, where strong currents over the sill are likely to pose a challenge for the eels to pass^8^,^9^ . Recently, Kettle^7^ and Capoccioni *et al.*^10^ have used modelling to conclude that it is likely that few, if any, of the Mediterranean eels reach the Sargasso Sea. However, observational data to support these models are limited: the only empirical study of eel migration in the Mediterranean Sea to date was carried out by Tesch^11^ in the 1980s. He used acoustic tags to track 12 eels for 1–6 days in the Alboran Sea; none of them crossed the Gibraltar Strait.

More recent technologies such as pop-up satellite tags (PSAT) and electronic data storage tags are now routine research tools that have opened up new possibilities for elucidating migration behaviour^12^. Recent tagging studies have tracked European eels from northern Europe well into the Atlantic Ocean, demonstrating their potential for tracking oceanic migrations^13^,^14^. These studies have also shown that, as they migrate towards the Sargasso Sea, eels undertake distinct diel vertical migration (DVM) behaviour with diurnal depth changes over several hundred metres closely related to sunset and sunrise. After sunset, eels occupy the upper mesopelagic layer (200–400 m) and at sunrise they descend into the lower mesopelagic layer (600–1000 m). The general vertical pattern has been confirmed in several other anguillid species^15^,^16^,^17^,^18^, suggesting this is a wide spread behavioural phenomenon.

The primary purpose of the present study was to investigate if European silver eels do indeed migrate from the Mediterranean Sea into the Atlantic Ocean. To achieve this, we attached PSAT tags to eight female silver eels caught during the autumn eel run in Southern France.

## Results

### Migration duration and distance

All eight tags transmitted data via the Argos system (Table 1); five transmitted from within the Mediterranean Sea and three from the Atlantic Ocean. Five tags surfaced prematurely; one tag surfaced 240 km from release after 29 days, three tags reported within the Mediterranean at distances of 636 to 1053 km from release and a fifth tag surfaced in the Atlantic, 1642 km from release. The remaining three tags surfaced as programmed, 6 months after release: two tags surfaced within the Atlantic Ocean, more than 2000 km from release, while one tag surfaced within the Mediterranean Sea, 719 km from release. In total, the tags yielded over 1000 days of temperature and depth time-series data.

### Migratory routes and travel speeds

**Figure 1:**
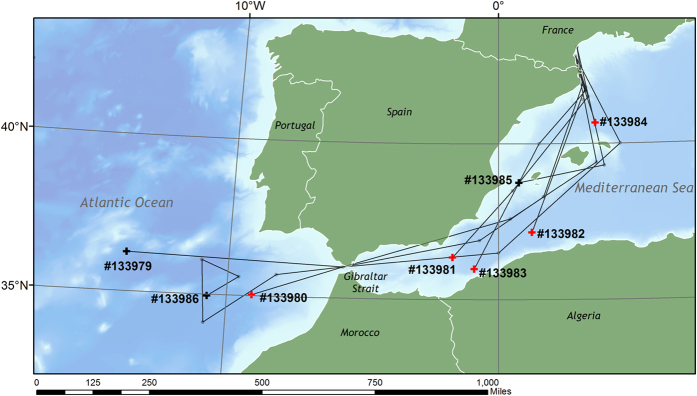
Map of western Mediterranean Sea and Eastern Atlantic Ocean, showing the approximate tracks of the individual tagged eels, based on a restricted number longitude and latitude estimates, see Methods for details. Crosses mark the pop-up position of each tag. Black crosses denote the tags that surfaced at the programmed date, while red crosses indicate the pop-up positions of tags attached to eels taken by predators. The map was drawn in Esri ArcMap 10.1, using GEBCO bathymetry ( http://www.gebco.net/) and ESRI map ( http://www.esri.com/software/arcgis) data.

After tagging and release, eels entered deeper water (>200 m) within 1–4 days (average 2.8 days). They initially followed a southern route away from the French coast turning west over time (Fig. 1). Based on the distance between waypoints identified from the data returned from the PSATs, net migration speed was calculated to range between 4.0 to 16.5 km d^−1^ in the Mediterranean and between 8.7 and 9.7 km d^−1^ in the Atlantic (Table 1). The two eels that entered the Atlantic Ocean reached the Gibraltar Straight after 77 (tag 133986), and 100 days (tag 133979), then continued into the Atlantic for over 600 km until the programmed day of release (Fig. 1). Although tag 133980 popped up in the Atlantic (Fig. 1), the depth and temperature data prior to crossing the Strait of Gibraltar were dissimilar to those reported by tags 133086 and 133979. This eel was probably eaten by a predator as it reached the Strait. Four of the five eels that did not escape the Mediterranean were also taken by predators, while one remained within the Mediterranean for the full duration of the study; this tag surfaced only 719 km from release.

### Vertical movements

**Figure 2:**
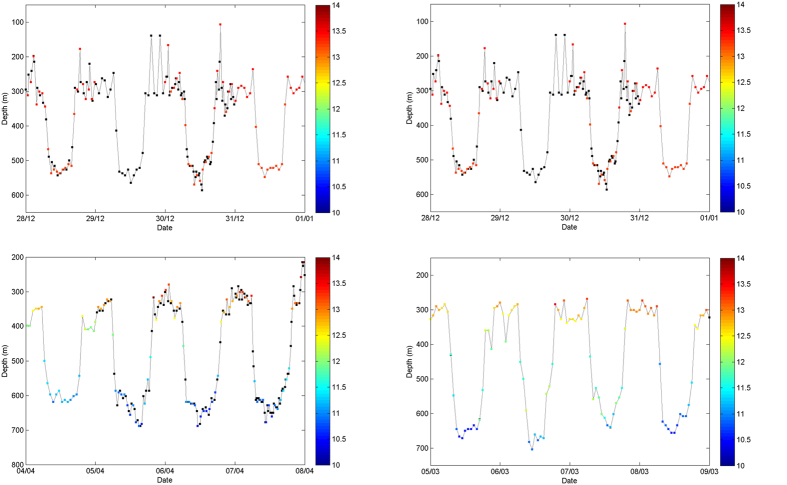
Sample plot of eels ID no. 133979 (left column) and 133986 (right column) showing a 4 day portion of vertical behaviour from the Mediterranean (upper panel) and the Atlantic Ocean (lower panel). Black symbols show depths where temperature data were not available due to variability in transmission conditions of the Microwave Telemetry X-tag; transmitted depth values may have no associated temperature value and vice versa.

**Figure 3:**
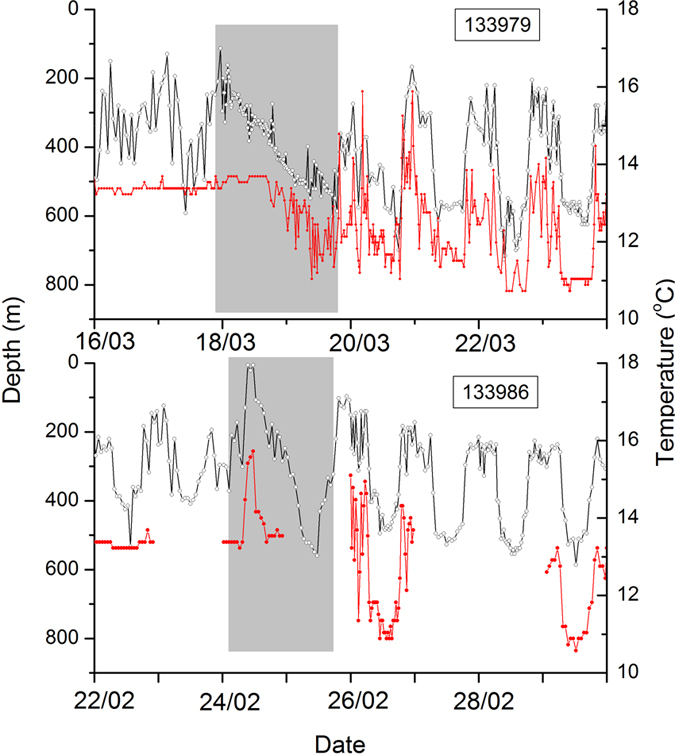
Recorded depth and temperature of eels while passing the Gibraltar Strait into the Atlantic Ocean (tags 133979 and 133986). The grey area shows the period from when the eel enters the Strait, based on the cessation of DVM, to the recurrence of DVM when the eel leaves the shelf slope and enters into the Atlantic Ocean. The grey symbols correspond to depth values and the red to the temperatures.

All eels established diel vertical migration (DVM) behaviour (typified in Fig. 2, see supplementary Fig. S1 for the time series for all eight eels) once they had entered deep water (>200 m), usually within a few days of release. In the Mediterranean, mean night-time depth was 371 m (range 446 to 254 m; Table 2), compared to day-time depths of 563 m (range 644 to 447 m). Despite these diel depth differences, the temperatures experienced by eels varied very little, with mean values during the night and day of 13.3 °C (Table 2). Both eels that passed the Gibraltar Strait (tags 133979 and 133986) showed a progressive descent during a period of 24 to 36 hours from 0–100 to 500–600 m depth (Fig. 3). After passing the Gibraltar Strait, the two eels quickly re-established DVM behaviour with mean depth at night and day of 350 m and 650 m respectively (Table 2). However, in contrast to the Mediterranean Sea, temperature variation was larger with mean values during the night and day of 12.6 and 10.8 ^o^C respectively (Table 2).

### Predation

**Figure 4:**
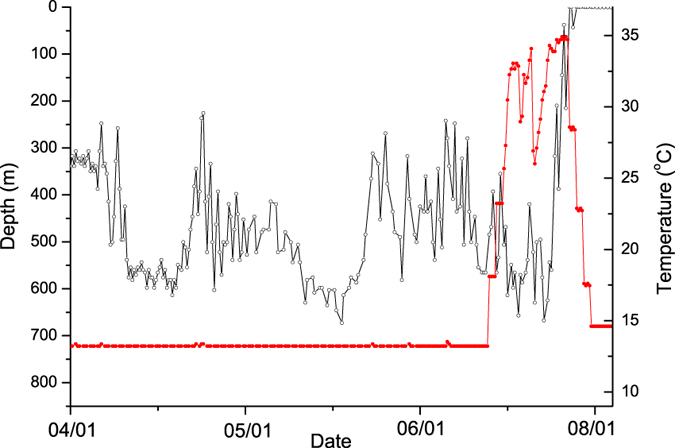
Example of predation (tag 133984). After predation (where temperature rises), the predator stays at depth for approximately 11 hours. Maximum recorded body temperature is 34.9 ^o^C. The grey symbols correspond to depth values and the red to the temperatures.

Five PSATs transmitted data prematurely (Table 1). All began transmitting due to the constant pressure release mechanism. In all five cases, the stereotypical DVM behaviour ceased abruptly, and was associated with a sudden change in recorded temperature, suggesting that predation was the likely cause. In one case (tag 133980), DVM behaviour ceased and depth changes became irregular. The light sensor did not record any light even in shallow water during daylight and the temperature data remained similar to the ambient temperature before predation, suggesting predation by an ectotherm (Table 1). In the other four cases, the temperature recorded by the tags rose from ambient temperatures of 13–14 ^o^C to more than 34 ^o^C, with several ascents to the surface over shorter periods and with no recorded light data transmitted (see supplementary Fig. S2 for the plots of predation event for the five eels concerned), indicating predation by endotherms. However, for one case in which recorded temperature rose above 34 °C (tag 133984), the depth record shows that the predator spent very long periods at depth between surfacing intervals (Fig. 4) including a period at depth of 11 h. No marine mammals have been observed to stay at depth for more than 2.3 h^19^. Although the evidence provided by the temperature data strongly indicates mammalian predation, it is improbable that the tag would fail to record a surfacing event, leaving the nature of the predator in this case somewhat uncertain.

## Discussion

Our results demonstrate, for the first time, that silver European eels from the Mediterranean Sea migrate towards and into the Atlantic Ocean according to *a priori* expectations based on life history^2^. Although the number of eels we tagged was relatively low, and our experiment was conducted solely with females, the results prove that eels from the Mediterranean are able to achieve the navigational demands of reaching the Atlantic Ocean and contribute to the Atlantic spawning migration with important consequences for eel management plans. In addition, we have shown that the behaviour of eels migrating from the Mediterranean is similar to that of migrating European eels from other regions^13^,^14^,^15^,^16^,^17^,^18^, underlining the importance of DVM in migration.

We used the same PSAT technology employed in other studies of eel migration^13^,^14^,^15^. As stated in those studies, we accept that the deployment of a satellite tag will increase drag and may disrupt behaviour, at least temporarily. Despite these effects, eels were able to achieve mean migration speeds of 10 km d^−1^ (4.0 to 16.5 km d^−1^ in the Mediterranean and 8.7 to 9.7 km d^−1^ in the Atlantic). This is similar, if slightly lower, to the speeds reported in other studies of European eel tagged with much smaller tags^20^,^21^ (mean 18.7 and 16 km d^−1^) , and also to the average speed of eels marked with the same tags but released in the Atlantic Ocean (mean 13.8 km d^−1^, ranging between 5 km to 25 km d^−1^)^13^. This difference may be related to the complexity of the currents found in Mediterranean Sea compared with the Atlantic Ocean^8^. While migration speed was slightly slower, the vertical movement behaviour was very similar to that of European eels reported in other tagging studies^11^,^13^,^14^,^21^. We are therefore confident that the results we have reported are representative of natural eel behaviour, while accepting that migration speeds and likelihood of predation may have been affected. Future miniaturization of PSATs will reduce the impact on eel behaviour.

The route of eels from the release site towards the Atlantic Ocean varied between individuals. Our reconstructions of migration were hampered by the uniform structure of the water column between 200 m and 700 m in the Mediterranean Sea, which had the consequence that the reconstructed trajectories were relatively simple and the finer details of small-scale meanders (and therefore total migration distance) was likely underestimated. However, despite this limitation, it was clear that all the eels migrated south-west in the direction of the Gibraltar Strait, and two eels escaped to the Atlantic during their time at liberty. Once out of the Mediterranean, one of the eels (tag 133986) performed a loop that may be related to a mesoscale eddy, commonly generated in the Mediterranean outflow^22^.

Despite the large distances that the eels travelled, even those that escaped to the Atlantic were still several thousand km from the assumed spawning area in the Sargasso Sea when the tags popped-up in June. Moreover, their measured migration speed was too low to arrive for the hypothesised April 1^st^ spawning event^1^. Although it is possible that eels would be able to take advantage of westward currents to assist the speed of their journey at some point^23^, eels escaping from the Mediterranean would presumably have to escape to sea significantly earlier to reach the Sargasso Sea by April 1^st^ (as suggested by Capoccioni *et al.*^10^) or the duration of the migration may be longer than previously accepted.

Timing and speed of migration is likely not the only critical factor in enabling eels to reach the Sargasso Sea. In five out of eight cases (63%), we recorded predation of the tagged eels. Marine mammals were likely responsible for at least three of the predation events. The same conclusion with similar data was shown in Wahlberg *et al.*^24^ and, for salmon, by Lacroix^25^. One of the tagged eels was evidently eaten by an ectotherm; post-predation dive patterns suggest it was likely a non-lamnid shark or similar sized fish^26^.

The threat of predation appeared to be reflected in the daily vertical migrations that the eels performed: eels stayed deep after dawn, and only moved into shallower water at dusk, a classic anti-predator strategy^27^ and in accordance with other oceanic studies of Anguillid eels^11^,^13^,^14^,^15^,^17^,^18^,^28^. However, unlike other studies, eels in the Mediterranean Sea did not experience any change in temperature over the course of this migration suggesting that thermal habitat occupation may be a consequence of, rather than a driver of, diel vertical migrations. Instead, the night time ascents into shallow water could be related to navigation to the Sargasso Sea^2^,^13^ or possibly a requirement for lower pressure to enable maturation of the gonads^16^,^29^. Notably, the stereotypical DVM disappeared when eels reached the Gibraltar Strait. This was likely to have been linked to the hydrography of the area^8^. Strong currents drive typically warmer saltier Mediterranean water into the Atlantic Ocean as a bottom current over the sill, while colder less salty Atlantic water flows into the Mediterranean Sea in the upper layers^9^. The continuous occupation of deep water and relatively deep descent suggest that the eels exploit the outgoing current from the Mediterranean Sea^8^ to reach the Atlantic. Once in the Atlantic Ocean, the stereotypical DVM returned, and the depth and temperature experience of the eels became similar to those reported by Aarestrup *et al.*^13^.

Our observations of European eels migrating from the Mediterranean do more than just add detail to our knowledge of eel natural history. The European eel is listed on Annex II of the Convention on International Trade in Endangered Species (CITES)^30^ because the recruitment of eels in Europe has declined dramatically since the 1980s. The causes of the decline are not fully clear, but appear to have impacts at each stage of the life-cycle^31^. To restore the stock to previous levels, the European Union has instructed every member state to draw up a comprehensive management plan with the goal of increasing the number of eels that escape to sea to reproduce^31^. Our study shows that female eels from the Mediterranean area are capable of migrating into the Atlantic Ocean and may, therefore, contribute to the spawning stock. While it is not currently possible to track male eels using the same technology to establish their migratory potential from the Mediterranean, our results from females nonetheless show that Mediterranean countries have an important role to play in contributing to conservation efforts to restore the eel population. This may be particularly important because eels at lower latitudes have a shorter generation time^1^. We advocate further studies from the Mediterranean Sea to investigate migration success and behaviour.

## Methods

### Tags

The PSATs used were the Microwave Telemetry X-tag ( http://www.microwavetelemetry.com/), which is 120 mm long, with a 185 mm long antenna. The maximum diameter of the float is 33 mm. Weight in air is 45 g and net buoyancy in water is approximately 0.025 ± 0.006 N, corresponding to a negative weight of 2.6 g. The tag measures and stores pressure, temperature and light data every two minutes. The time of release of PSATs was set to 6 months after deployment, the hypothesized time required for migration to the spawning area^1^. The constant pressure release feature that detaches the tag if the depth reading remains within 3 m for a period of four days was deactivated for the first 20 days after deployment to avoid premature release associated with limited movements in shallow water. After a tag pops up, a subset of the stored time-series of depth and temperature is transmitted from the tag to low earth orbiting Argos satellites, where it can be downloaded. The transmitted dataset has a 15 minute sampling period but, depending on the transmission conditions, there can be gaps of up to one hour between transmitted observations. Additionally, due to the way that data are dissembled before transmission, depth values may have no associated temperature value and vice versa. The temperature measurement range is −4 to 40 ^o^C with a resolution of 0.23 ^o^C. The depth range is 0 to 1300 m with a resolution varying between 0.34 and 5.4 m depending on the gain value, which is automatically selected according to the depth measured at midnight each day. More details on how data from the X-tag is coded and transmitted can be found at http://microwavetelemetry.com/fish/understanding_data_xtag.cfm.

### Tagging method

The eels used in the tagging experiment were captured by commercial fishermen in the lagoons of Salses-Leucate and Gruissan, France, during the authorized fishing season. Procedures were conducted in accordance with the guidelines of the French Ethical Committee for animal experiments. Tagging was conducted by trained and licensed scientists working under the authority and approval of the certificat d’experimenter sur les animaux vertébrés vivants (experimental animal certificate) number 66.0801 (Elisabeth Faliex) of the CEFREM, University of Perpignan and number 2012-DY-2934-00007 (Kim Aarestrup) of DTU, Denmark. All eels selected for tagging were females because even the largest male eels are too small for a PSAT attachment. A total of eight eels were tagged, with a mean length of 94.0 ± 43.6 cm (range 86.5 cm - 99.8 cm) and weighing between 1.5 kg to 2.6 kg in weight (average 1.9 ± 0.4 kg). Silver index was calculated for each eel based on the Pankhurst’s ocular index (OI) as follows:


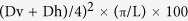


where Dv and Dh are the vertical and horizontal eye diameters and L is the size of the eel (all measured in mm). All eels in this study had OI ≥ 6.5 and can be considered as silver individuals ready to migrate^32^ (Table 1).

Before tagging, eels were anaesthetised using Aqui-S® (Aqua-S, New Zealand) at a concentration of 600 mg/L. The tags were attached using stainless steel wires in a 3-point attachment inserted dorsally under the skin (for details see^33^). The rationale behind this attachment technique is that the force exerted by the tag on the eel is concentrated on the anterior attachment point allowing the two posterior attachment points to heal after tagging. If the anterior loop is rejected the tag is still attached by the two remaining loops. In long-term tests, this attachment method was successful in retaining the tag for more than 6 months in 50% of the cases, with the first loss recorded after 4 months (n = 8)^33^.

Tagged eels were held in covered tanks for one day to allow for recovery. There were no post-tagging mortalities during this recovery period. The eels were then transported to the outlet of the lagoons of capture in oxygenated tanks, transferred into a boat and released offshore, app. 200 m from the lagoon outlet of Salses-Leucate (42°47′51.9″N ; 3°02′35.8″E) and Gruissan (43°05′38.5″N ; 3°06′53.5″E).

### Determining the fate of eels

Since all PSAT tags rise to the surface once they become detached, the data preceding this terminal point were assessed to determine the fate of their host. When external tags were attached for the expected deployment duration (6 months), it was assumed that the entire record was representative of eel behaviour. However, some tags detached earlier than expected. In these cases, the recorded regular diurnal pattern of depth, temperature or light typically changed abruptly mid-record, indicating predation.

### Analysing migrations

Due to the migration depth of the eels (>200 m), the onboard daylight geolocation system of the PSAT gave no meaningful data to enable light-based estimates of eel location. Instead, frequent estimates of location were made using the depth and temperature time-series recorded by the tags. First, we assumed that significant diurnal changes in swimming depth of the eels was a proxy for daylight change that were cued to dawn and dusk. This assumption is substantiated by the observation that, in eels tracked with archival tags in the Baltic, the times of beginning and end of a daily resting period at the seafloor coincided closely with the time of civic twilight at the date and longitude of the eels^21^. The mean of the dawn (significant descent) and dusk (significant ascent) times in UTC enables measurement of the time of local noon. The difference between 1200 UTC and local noon gives the longitude after correction for the difference between apparent solar time and mean solar time. Data recovered from PSATs has a 15 minutes sampling rate at best, so a Fast Fourier transform was used to smooth the data. The smoothed time series were then used to find the times when the eel crossed three different and well separated depth thresholds, and the mean midpoint of these times of local noon was calculated and used to estimate the longitude (as for Westerberg *et al.*^14^).

The time series of longitude estimates were then used as a starting point for further reconstruction of the trajectories of the eels. The bathymetry of the general area and the maximum depth recorded each day gave a restriction on the possible latitude. An easily defined checkpoint was the passage through the Strait of Gibraltar, when the eel passed from vertically homothermal to clearly stratified water on the Atlantic side. From this point on, a second restriction was given by the water temperature at the depths occupied by the eel during day and night. This was compared to oceanographic data using the operational IBI (Iberian Biscay Irish) Ocean Analysis and Forecasting system. The system is based on a (eddy-resolving) NEMO model application run at 1/36° horizontal resolution. Daily modelling data were analysed with the program Ocean Data View 4 34.

For speed calculation, the beginning of the migration was defined as the day and position of release. The end point of the migration was taken as the pop-up position when the tag was released on the programmed date. For premature pop-up the transmission was delayed until the constant pressure mechanism was actuated. In those cases the first position was corrected for drift during the delay using an extrapolation of the average daily drift (direction, velocity) observed after the first transmission. The end positions of eels that were eaten by predators were uncertain due to the unknown post-predation movement of the predator. We considered the passage of the Strait of Gibraltar sill (taken here as 36.00 N and 5.77 W) to occur when the descent phase was continuous over more than a day-night cycle and without an accompanying up-down migration.

Mean night and day-time depth and temperature were calculated during the oceanic portion of the migration. The beginning of active oceanic migration was defined as the day where the maximum depth reached by the eel exceeded 200 m. In cases where the eel may have been taken by a predator, depth and temperature data were included until the final day on which the stereotypical diurnal migrations were recorded.

## Additional Information

**How to cite this article**: Amilhat, E. *et al.* First evidence of European eels exiting the Mediterranean Sea during their spawning migration. *Sci. Rep.* **6**, 21817; doi: 10.1038/srep21817 (2016).

## Supplementary Material

Supplementary Information

## Figures and Tables

**Table 1:** Biological and migration metrics from the eight PSAT tagged eels.

Tag ID#	Length (cm)	Weight (kg)	Ocular index	Release date	Pop up date	Duration (d)	Distance (Km)	Net migration Speed (km d^−1^) (Med/Atl)	Fate/ max T°
133979	95.2	1.62	14.3	09-12-2013	09/06/2014	182	2157	14.5/8.7	Pop
133980	90.8	2.09	13.5	09-12-2013	22/03/2014	102	1642*	*	S/16
133981	93.4	1.63	10.7	09-12-2013	03/03/2014	84	1053	12.5/	W/37.3
133982	96.0	1.85	10.5	09-12-2013	27/04/2014	139	636	4.6/	W/35.3
133983	99.8	2.63	12.7	09-12-2013	24/03/2014	105	992	9.4/	W/36.8
133984	86.5	1.46	12.9	09-12-2013	06/01/2014	29	240	8.3/	U/34.9
133985	98.7	2.24	12.4	10-12-2013	09/06/2014	181	719	4.0/	Pop
133986	91.8	1.68	14.6	10-12-2013	09/06/2014	181	2296	16.5/9.7	Pop

^*^Eel was probably taken by a predator in or near the Strait of Gibraltar and the track in the Atlantic is mainly of the predator.

Metrics are indicated as well as migration duration, distance travelled, net migration speed (in the Mediterranean and Atlantic, respectively) and suggested ultimate fate indicated by letters (S = Shark, W = Whale, U = Unknown) and the maximum temperature (max T°) in the hours after predation. “Pop” indicates that the tag was attached to the eel until the programmed day of release.

**Table 2:** Habitat occupation of eels.

Tag	Mediterranean Sea			Atlantic Ocean		
	Days in Mediterranean	Day temp/ night temp (C)	Day depth/ night depth (m)	Days in Atlantic	Day temp/ night temp (C)	Day depth/ night depth (m)
133979	97	13.3/13.4	526.5/269.8	81	10.9/12.6	647.3/355.4
133980_p	85	13.3/13.4	552.5/437.7	-	-	-
133981_p	78	13.3/13.4	610.0/429.4	-	-	-
133982_p	122	13.2/13.2	596.6/376.5	-	-	-
133983_p	98	13.2/13.2	625.2/370.1	-	-	-
133984_p	26	13.2/13.2	502.0/382.8	-	-	-
133985	176	13.3/13.3	643.6/445.7	-	-	-
133986	72	13.3/13.4	447.0/254.0	105	10.6/12.6	651.2/345.1
Average	94.3	13.3/ 13.3	562.9/370.8	93	10.8/12.6	649.6/350.2

Values are averages of temperature and depth during the oceanic portion of the migration (when eel occupies water depth >200 m). p denotes that the eel suffered predation.
