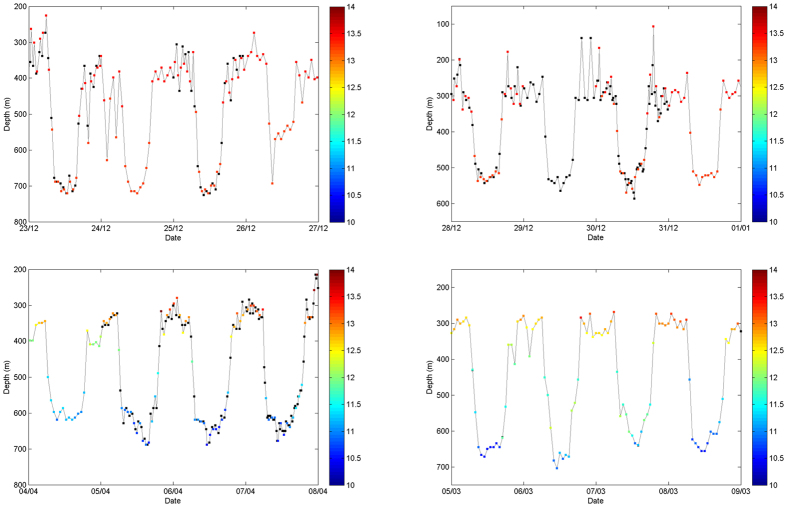# Corrigendum: First evidence of European eels exiting the Mediterranean Sea during their spawning migration

**DOI:** 10.1038/srep26214

**Published:** 2016-05-20

**Authors:** Elsa Amilhat, Kim Aarestrup, Elisabeth Faliex, Gaël Simon, Håkan Westerberg, David Righton

Scientific Reports 6: Article number: 21817; 10.1038/srep21817 published online: 02242016; updated: 05202016.

In Figure 2 of this Article, the upper left graph is a duplication of the upper right graph. The correct Figure 2 appears below as Figure 1.

## Figures and Tables

**Figure 1:**