# Supplementary material for: First evidence of European eels exiting the Mediterranean Sea during their spawning migration
Source: Sci Rep. 2016 Feb 24;6:21817. doi: 10.1038/srep21817 (PMC4764813; doi:10.1038/srep21817)
Supplement: Supplementary Information [file srep21817-s1.pdf]

Supplementary information

First evidence of European eels exiting the Mediterranean Sea during their spawning migration

Elsa Amilhat [1,2*], Kim Aarestrup[3], Elisabeth Faliex [1,2], Gaël Simon[1,2], Håkan Westerberg[4], and David Righton[5]

Supplementary Figure S1: Time-series (depth in m and temperature in °C) transmitted by tags deployed on European eels in the Mediterranean. Plots show depth data coloured by temperature. Grey points indicate depth measurements transmitted without a concurrent temperature record.

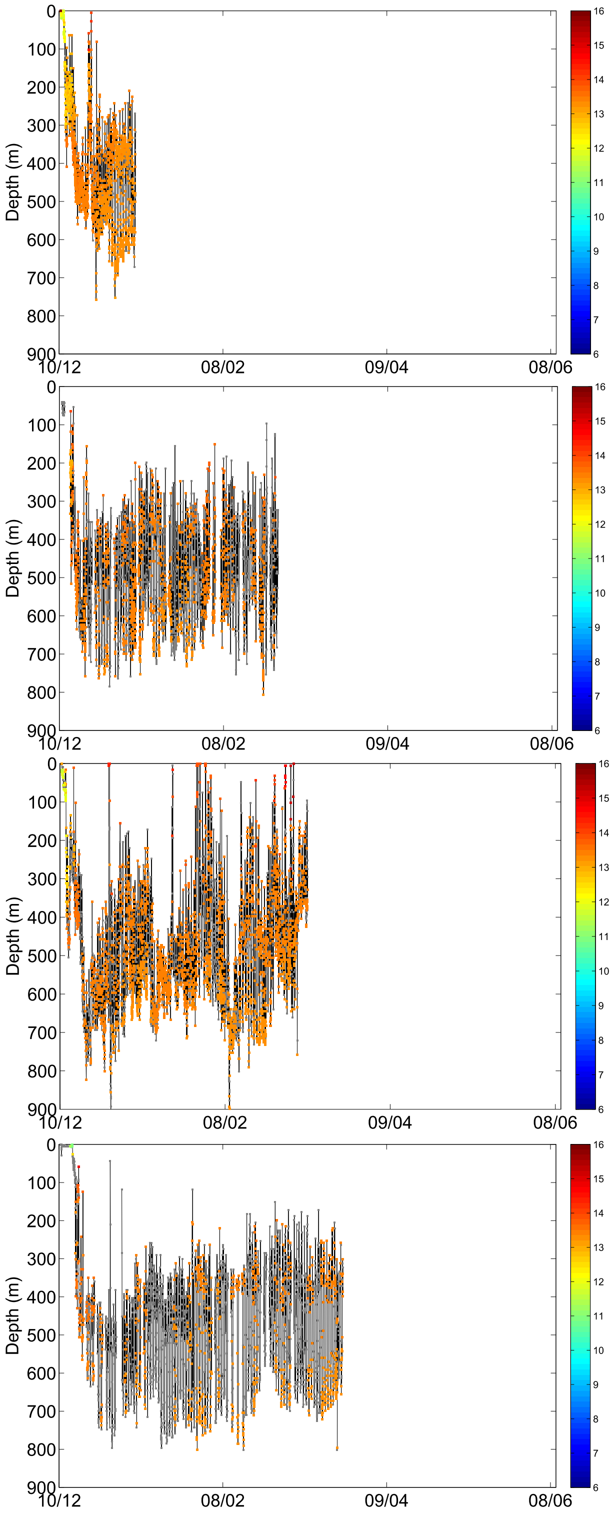

133984

133981

133980

133983

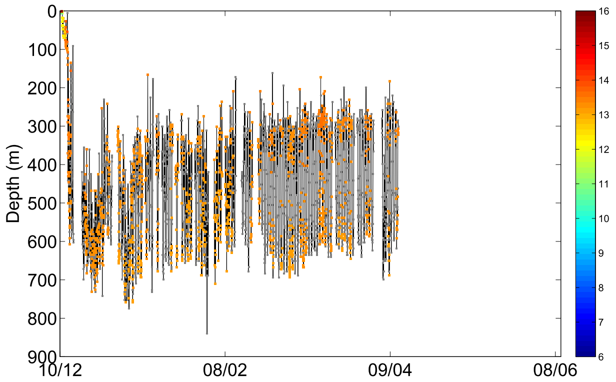

133982

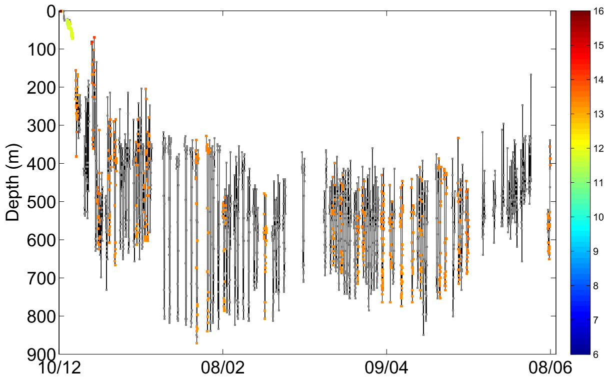

133985

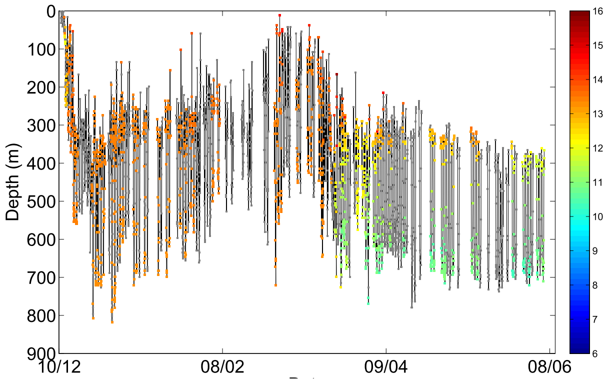

133979

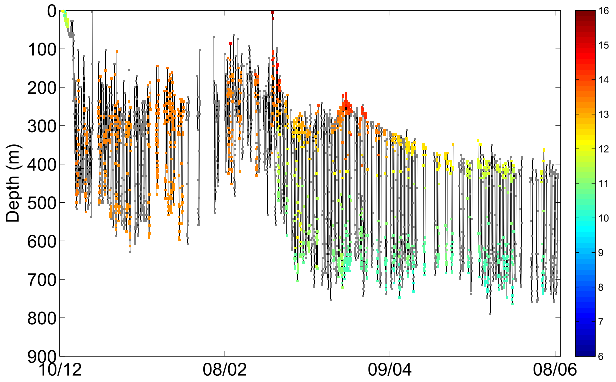

133986

Supplementary Figure S2: Plots of predation events. The grey rectangles indicate when the predation took place. The grey symbols correspond to depth values and the red to the temperatures.

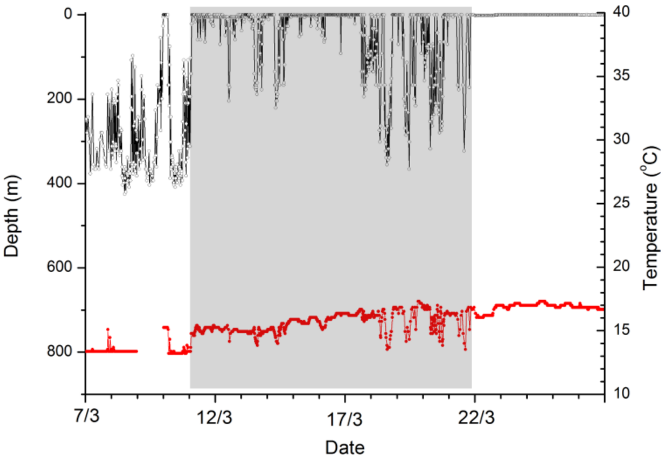

133980

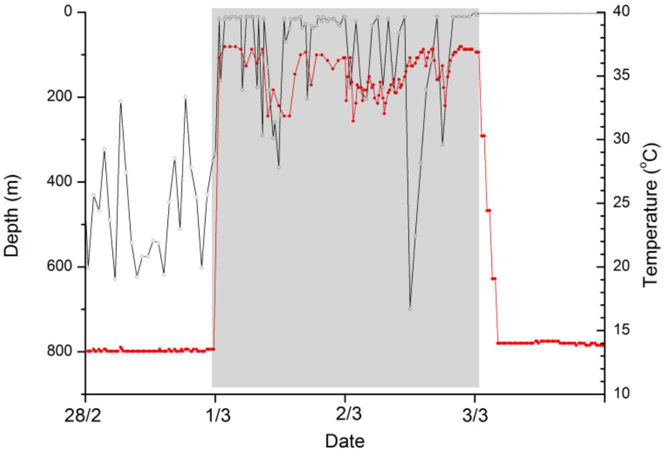

133981

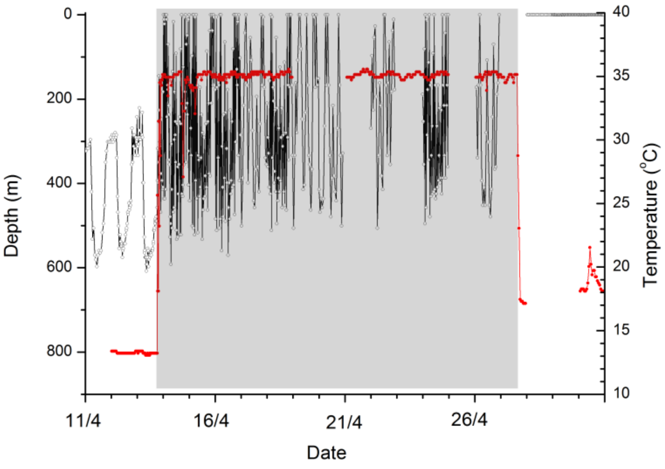

133982

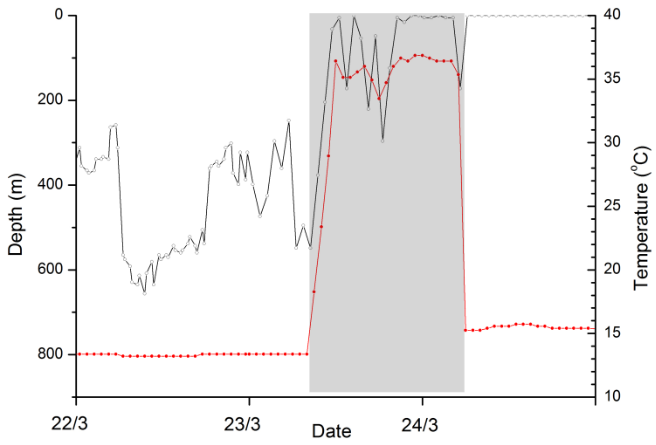

133983

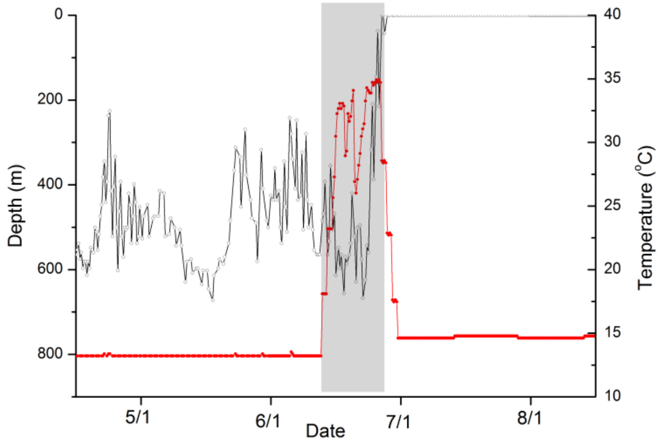

133984
